# Identification of drug combinations administered by continuous subcutaneous infusion that require analysis for compatibility and stability

**DOI:** 10.1186/s12904-017-0195-y

**Published:** 2017-03-23

**Authors:** Andrew Dickman, Matthew Bickerstaff, Richard Jackson, Jennifer Schneider, Stephen Mason, John Ellershaw

**Affiliations:** 1 Marie Curie Palliative Care Institute Liverpool, Cancer Research Centre, Liverpool, L3 9TA UK; 2 Cancer Research UK Liverpool Cancer Trials Unit, University of Liverpool, Liverpool, L69 3GL UK; 3 School of Biomedical Sciences and Pharmacy, Faculty of Health and Medicine, University of Newcastle, Newcastle, Australia

**Keywords:** Palliative medicine, Drug combinations, Infusions, Subcutaneous, Delphi technique, Surveys, Questionnaires, Patient safety

## Abstract

**Background:**

A continuous subcutaneous infusion (CSCI) delivered via syringe pump is a method of drug administration used to maintain symptom control when a patient is no longer able to tolerate oral medication. Several classes of drugs, such as opioids, antiemetics, anticholinergics, antipsychotics and benzodiazepines are routinely administered by CSCI alone or in combinations. Previous studies attempting to identify the most-common CSCI combinations are now several years old and no longer reflect current clinical practice. The aim of this work was to review current clinical practice and identify CSCI drug combinations requiring analysis for chemical compatibility and stability.

**Methods:**

UK pharmacy professionals involved in the delivery of care to palliative patients in hospitals and hospices were invited to enter CSCI combinations comprised of two or more drugs onto an electronic database over a 12-month period. In addition, a separate Delphi study with a panel of 15 expert healthcare professionals was completed to identify a maximum of five combinations of drugs used to treat more complex, but less commonly encountered symptoms unlikely to be identified by the national survey.

**Results:**

A total of 57 individuals representing 33 separate palliative care services entered 1,945 drug combinations suitable for analysis, with 278 discrete combinations identified. The top 40 drug combinations represented nearly two-thirds of combinations recorded. A total of 23 different drugs were administered in combination and the median number of drugs in a combination was three. The Delphi study identified five combinations for the relief of complex or refractory symptoms.

**Conclusion:**

This study represents the first step towards developing authoritative national guidance on the administration of drugs by CSCI. Further work will ensure healthcare practitioners have the knowledge and confidence that a prescribed combination will be both safe and efficacious.

## Background

A continuous subcutaneous infusion (CSCI) is a method of drug administration used to maintain symptom control when a patient is no longer able to tolerate oral medication. A syringe pump (also referred to as a syringe driver) is used to deliver a CSCI, which is considered fundamental for continued symptom management in palliative care [1]. Several classes of drugs, such as opioids, antiemetics, anticholinergics, antipsychotics and benzodiazepines are routinely administered by CSCI alone or in combinations [2].

**Fig. 1:**
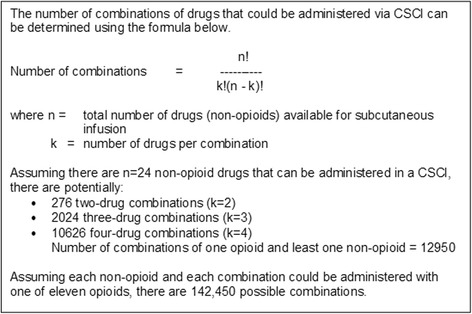
Calculation of number of possible drug combinations. Adapted from reference [35]

There have been several national surveys that have attempted to identify commonly used mixtures [3–6]. These studies, however, are several years old and do not reflect current practice. The most recent survey of CSCI use in the UK was performed over a decade ago in 2004 [6]. The majority of combinations contained either two or three drugs (44 and 30% respectively) and an opioid was invariably a component. The authors concluded that compatibility and stability data were unavailable for more than half of the frequently used combinations. This is unsurprising for two reasons. Firstly, laboratory analysis is expensive and laborious. Secondly, the number of potential combinations is vast; Dickman et al. [2] identified 35 drugs that could be administered by CSCI, 11 of which were opioids. Based on these figures, there are theoretically 142,450 combinations (Fig. 1). Of course, certain combinations of drugs are impractical, so the number of clinically useful combinations is significantly lower. For example, for five non-opioids and one opioid, there are potentially 56 combinations comprising up to five drugs.

Parenteral administration of drugs is a recognised cause of medication error, and compatibility and stability of drug combinations is an issue of patient safety [7]. Despite widespread application in palliative care, there remain only three major sources of compatibility and stability information relating to CSCIs [2, 8, 9]. These reference sources provide information mainly about the visual compatibility of drug combinations, often relying on a clinical assessment to determine whether or not a particular combination is suitable for administration by CSCI. While many mixtures may appear physically compatible (i.e. clear, colourless and free from precipitation) the risk of chemical incompatibility cannot be ignored. There is a clear need to identify compatibility and stability of drug combinations administered by CSCI, especially those combinations most commonly prescribed. The aim of this project was to review current clinical practice to identify drug combinations that require analysis for chemical compatibility and stability.

## Methods

In order to identify a list of drug combinations that would be subject to subsequent chemical analysis, two methods were employed: a national survey of practice and a Delphi study. This work was registered with the Liverpool Clinical Trials Unit (LCTU), which provided structured data monitoring, assessment and logistical support. Members of the Association of Supportive and Palliative Care Pharmacy (formerly Palliative Care Pharmacists’ Network) were approached for both methods.

### National survey

The national survey invited UK pharmacists and pharmacy technicians involved in the delivery of care to palliative patients in hospital and hospices to take part. Over a 12-month period, participants were asked to contribute to an anonymous web-based database that recorded information pertaining to CSCIs comprising two or more drugs used for symptom management of patients aged 18 years and above during the last months or days of life. Participants entered information into the database about drug(s), dose(s), diluent used, total volume of infusion, duration of infusion and visual compatibility. The web-based system, designed and administered by LCTU, ensured patient anonymity while preventing duplication of entry. The database was analysed and a list describing the most frequent 40 combinations was produced. A literature search was then performed to determine if any the top 40 combinations identified by this national survey had already undergone chemical analysis.

### Delphi study

In addition to the national survey, a Delphi study was undertaken using an online web questionnaire, hosted by www.smartsurvey.co.uk. The purpose of the Delphi study was to identify a maximum of five combinations of drugs used to treat more complex symptoms that required investigation for compatibility to improve the quality of patient care. This approach was deemed necessary as combinations prescribed for more difficult, but less commonly encountered symptoms, would not be identified by the national survey. The panel of experts in this study comprised 15 healthcare professionals split evenly between doctors, nurses and pharmacists. Each expert was required to have at least 5 years’ palliative care clinical experience and be involved in the prescribing of, setting up of, or checking of CSCIs.

Pharmacists were contacted and asked if there was an interest to participate in this study, provided they met the criteria above. They were also requested to nominate senior medical and nursing staff at their place of work who met the criteria and were willing to participate. Experts were selected based on promptness of response to agree to participate. One week before disseminating each questionnaire, all the experts received a pre-notification personalised email sent by the researcher. All further communication between researcher and respondents was indirect as the host website served as a conduit, ensuring respondent anonymity. All participants gave informed consent before attempting each questionnaire. The website hosting the survey anonymised the questionnaires, preventing identification of respondents by the researcher.

In the first-round of the Delphi study, the experts were asked to identify up to 10 combinations of drugs that had been used, or would have been used if there were compatibility data, for the management of complex or refractory symptoms. The experts were allowed complete freedom with their responses. A second literature search was undertaken to determine if any of the most common 25 combinations identified in the first-round of the Delphi study had already undergone chemical analysis. In the second round, the experts were required to select and rank ten combinations from the list of the 25 most common combinations of drugs identified in the first-round. In the final round, the experts were presented with the top 15 combinations derived from the second round and asked to rank them. Due to the potential for variability of responses, consensus agreement was based on the mean rank order. The top five drug combinations after three rounds were selected.

## Results

A total of 57 individuals representing 33 separate palliative care services completed the national survey. At the time the database was locked, 1,945 combinations had been recorded (2,132 samples were collected, but 186 were excluded due to erroneous entry), with 278 discrete combinations of drugs being identified. Analysis of the database revealed 23 different drugs had been administered in combination by CSCI, six of which were opioids (see Table 1). The four most frequently prescribed drugs (midazolam, morphine, oxycodone and levomepromazine) accounted for 52% of all administered drugs. The median number of drugs administered by CSCI was three, with 50.6% of combinations comprising three or more drugs. Almost all CSCIs were administered over a duration of 24 h (1915/1945; 98.5%) with Water for Injections (WFI) being the most commonly used diluent (1346/1945; 69.2%). An opioid was present in 91.5% of the combinations.

**Table 1:** Frequency and doses of drugs that appeared in the database

Drug	Frequency	1^st^ quartile dose (mg)	3^rd^ quartile dose (mg)	Mean dose (mg)	Median dose (mg)
Midazolam	1002	10	20	16.6	10.0
Morphine	568	10	40	32.0	20.0
Oxycodone	549	15	60	52.5	30 .0
Levomepromazine	514	6.25	25	22.0	12.5
Haloperidol	492	2.5	3	3.0	3.0
Alfentanil	359	2	14	37.8	5.0
Hyoscine butylbromide	330	60	120	86.9	80.0
Diamorphine	289	10	80	77.7	30.0
Metoclopramide	276	30	60	49.8	40.0
Cyclizine	171	150	150	143.4	150.0
Clonazepam	165	0.5	2	1.72	1.0
Glycopyrronium	116	0.6	1.2	1.13	1.2
Dexamethasone	93	0.5	16	1.73	8.0
Octreotide	56	0.3	1	0.5	0.6
Ketamine	30	60	350	220.2	175.0
Ondansetron	21	8	20	14.5	12.0
Granisetron	12	2	4	3.25	3.5
Methadone	12	35	60	44.8	52.5
Hyoscine hydrobromide	8	0.6	1.6	1.3	1.4
Ketorolac	5	60	60	60.0	60.0
Hydromorphone	4	70	90	80.0	80.0
Ranitidine	4	150	225	162.8	150.0
Diclofenac	1	75	75	75.0	75.0

The top 40 represented nearly two-thirds of the 1,945 combinations recorded (see Table 2), comprising 20 two-drug, 19 three-drug and 1 four-drug combinations. Compatibility and stability data at clinically relevant doses were available for less than one third of the top 40 combinations [10–21].

**Table 2:** Top 40 combinations present in the 1,945 recorded CSCIs

Drug Combination				Frequency
Drug 1	Drug 2	Drug 3	Drug 4	
Morphine	Midazolam	-	-	124^a^
Oxycodone	Midazolam	-	-	81^a^
Oxycodone	Levomepromazine	Midazolam	-	60
Morphine	Metoclopramide	-	-	57
Diamorphine	Midazolam	-	-	55^a^
Morphine	Levomepromazine	Midazolam	-	53
Oxycodone	Haloperidol	Midazolam	-	52
Oxycodone	Haloperidol	-	-	49^a^
Morphine	Haloperidol	Midazolam	-	46
Alfentanil	Haloperidol	-	-	36
Oxycodone	Levomepromazine	-	-	35^a^
Alfentanil	Midazolam	-	-	35
Alfentanil	Levomepromazine	Midazolam	-	34
Morphine	Haloperidol	-	-	29
Oxycodone	Metoclopramide	-	-	29^a^
Oxycodone	Haloperidol	Hyoscine butylbromide	-	29^a^
Morphine	Cyclizine	-	-	28
Morphine	Levomepromazine	-	-	22^a^
Alfentanil	Haloperidol	Midazolam	-	22^a^
Diamorphine	Metoclopramide	-	-	21^a^
Alfentanil	Metoclopramide	-	-	21
Alfentanil	Hyoscine butylbromide	Levomepromazine	-	20
Oxycodone	Metoclopramide	Midazolam	-	20
Diamorphine	Cyclizine	-	-	19^a^
Diamorphine	Haloperidol	Midazolam	-	19
Oxycodone	Hyoscine butylbromide	Midazolam	-	18
Alfentanil	Hyoscine butylbromide	Levomepromazine	Midazolam	18
Alfentanil	Metoclopramide	Midazolam	-	17
Morphine	Hyoscine butylbromide	Midazolam	-	17
Diamorphine	Levomepromazine	-	-	17^a^
Diamorphine	Levomepromazine	Midazolam	-	17
Morphine	Haloperidol	Hyoscine butylbromide	-	17
Morphine	Metoclopramide	Midazolam	-	16
Morphine	Glycopyrronium	Midazolam	-	15
Alfentanil	Cyclizine	-	-	14
Oxycodone	Haloperidol	Hyoscine butylbromide	-	14
Diamorphine	Haloperidol	-	-	14^a^
Morphine	Hyoscine butylbromide	-	-	13
Dexamethasone	Ketamine	-	-	13
Diamorphine	Cyclizine	Midazolam	-	13

^a^ combinations identified as being analysed at clinically relevant doses for compatibility and stability [10–21]; note that all analysed combinations were reported as compatible under stated conditions

The e-Delphi study was successfully completed by all 15 healthcare professionals, enabling the identification of five combinations used for complex or refractory symptoms that required analysis for compatibility and stability (see Table 3). No compatibility and stability data were found for these combinations. Only one of the five combinations appeared in the survey database (oxycodone, glycopyrronium and levomepromazine) and all comprised three drugs.

**Table 3:** Top 5 combinations identified by consensus from the e-Delphi study

Combination			Mean rank score
Drug 1	Drug 2	Drug 3	
Alfentanil	Hyoscine butylbromide	Octreotide	4.46
Oxycodone	Hyoscine butylbromide	Octreotide	6.00
Oxycodone	Glycopyrronium	Midazolam	6.53
Morphine	Dexamethasone	Ranitidine	8.33
Oxycodone	Ketamine	Levomepromazine	9.13

## Discussion

This survey represents the most recent analysis of drug combinations administered by CSCI in the UK. The most commonly prescribed drug administered by CSCI was midazolam, while the most commonly prescribed opioid was morphine. Diamorphine was identified as the least commonly prescribed opioid. The previous national survey, however, identified diamorphine as the opioid most commonly administered by CSCI [6]. The worldwide shortage of diamorphine during the winter of 2004 forced services to adopt morphine as the opioid of choice and it would appear that many have not reverted to diamorphine. In 2012, the National Institute for Health and Clinical Excellence recommended that the parenteral opioid with the lowest acquisition cost (i.e. morphine) should be used first-line [22]. Diamorphine, with its superior solubility, appears to be generally reserved for patients with a relatively high opioid requirement, as reflected by mean and median doses of 77 mg and 30 mg respectively (Table 1).

A previous literature review found that only 70 drug combinations suitable for administration by CSCI had been investigated for compatibility and stability. The majority of these (88.6%) referred to two-drug combinations [23]. Indeed, a review of the literature for the top 40 combinations identified by the national survey revealed that only 13 (32.5%) have been analysed at clinically relevant doses for compatibility and stability, the majority of which comprised two drugs. The five combinations identified by the Delphi study were those mainly associated with the management of difficult or recalcitrant symptoms. Such combinations were expected to occur infrequently, a supposition vindicated by the appearance of only one combination in the database derived from the survey. These combinations often reflect specialist practice. It was unclear from the survey which area of practice the data were collected. A survey of specialist palliative care inpatient services only would be expected to identify a different list of commonly used combinations. Indeed, analysis of practice at a specialist palliative care inpatient unit revealed that 70% of the combinations comprised three or more drugs (compared to 50.6% in this survey) [24]. Since the majority of currently available compatibility and stability data refer to two-drug combinations, there is a disparity between available evidence and clinical practice.

A limitation of the Delphi technique is the risk of selection bias often associated with the choice of expert panel. Indeed, there are no universally accepted criteria for the selection of experts using the Delphi technique [25]. Selecting panel members through methods other than acquaintance is believed to minimise bias [26, 27]. In an attempt to reduce the risk of researcher bias, experts were selected for the Delphi study through a mix of promptness of response to the call to participate and snowballing. Both methods, however, may be associated with a potential for bias. A promptness to respond to the invitation and a willingness to participate suggests the expert has an interest in the subject under scrutiny, which carries a risk of self-selection bias. It has been proposed that there is an inherent bias in Delphi studies because experts may volunteer to take part as they hold particular views about the subject matter, which could influence the consensus process [28]. Nonetheless, it has been shown that those who are willing to participate in expert panels are representative of their unwilling colleagues [29]. The ‘snowballing’ technique is a conventional recruitment method in Delphi research [30–32]. This technique, however, could have led to selection bias because of its unrepresentative approach by potentially limiting the breadth of experience and geographical distribution of participants [33, 34].

## Conclusion

Further work based on the outcomes of this study will ensure that healthcare practitioners will have the knowledge and confidence that a prescribed combination can be safely administered and that the expected therapeutic goal will be achieved. Given the dispersion of doses within the dataset for each drug, analysis utilising the first and third quartiles are recommended for any future compatibility tests. This study represents the first step towards developing an authoritative national guide which will inform healthcare professionals about the administration of drugs by a CSCI.

## Abbreviations

CSCI: Continuous subcutaneous infusion
UK: United Kingdom
WFI: Water for injections
